# Immune Checkpoint Inhibitor-Associated Colitis and Hepatitis

**DOI:** 10.1038/s41424-018-0049-9

**Published:** 2018-09-19

**Authors:** Haritha G. Reddy, Bryan J. Schneider, Andrew W. Tai

**Affiliations:** 1 Rogel Cancer Center, Division of Hematology/Oncology, Department of Internal Medicine, University of Michigan, Ann Arbor, MI USA; 2 Division of Gastroenterology, Department of Internal Medicine, University of Michigan, Ann Arbor, MI USA; 3 Department of Microbiology & Immunology, University of Michigan, Ann Arbor, MI USA; 4 Medicine Service, VA Ann Arbor Healthcare System, Ann Arbor, MI USA

## Abstract

Immune checkpoint inhibitors (ICPIs) are monoclonal antibodies that target downregulators of the anti-cancer immune response: cytotoxic T-lymphocyte antigen-4, programmed cell death protein-1, and its ligand PD-L1. ICPIs are now approved for the treatment of a wide array of malignancies, with rates of durable responses in the metastatic setting far exceeding what would be expected from conventional chemotherapy. ICPIs have also been associated with rare but serious immune-related adverse events due to over-activation of the immune system that can affect any organ, including the gastrointestinal tract and liver. As the use of ICPIs in oncology continues to increase, ICPI-associated colitis and hepatitis will be encountered frequently by gastroenterologists and hepatologists. This review will focus on the diagnosis and management of ICPI-associated colitis and hepatitis. We will also compare these ICPI-related toxicities with sporadic inflammatory bowel disease and autoimmune liver disease.

## Introduction

Cancer is the second leading cause of death in the United States, accounting for nearly one out of every four deaths. Over the past 30 years, significant improvements in time to diagnosis and treatment have increased the 5-year survival rate for all cancers^1^. A recent breakthrough in oncology has been the advent of immune checkpoint inhibitors (ICPI); monoclonal antibodies that target important downregulators of the anti-cancer immune response: cytotoxic T-lymphocyte antigen-4 (CTLA-4), programmed cell death protein-1 (PD-1), and its ligand (PD-L1). CTLA-4 functions as a negative regulator of T-cell activity and is expressed on the surface of CD4 and CD8 positive T-cells and on subsets of B-cells and thymocytes^2^. Similarly, PD-1 is a receptor found on monocytes, T cells, B cells, dendritic cells, and tumor-infiltrating lymphocytes. PD-1 binds to PD-L1, which may be overexpressed on tumor cells and antigen-presenting cells, suppressing T-cell receptor signaling and responses^3^. CTLA-4 inhibition with ipilimumab is thought to block the initial steps of T-cell activation and proliferation within lymph nodes, whereas PD-1/PD-L1 inhibitors (nivolumab, pembrolizumab, atezolizumab, avelumab, and durvalumab) target T cells at a later stage of the immune response within the tumor and peripheral tissues^4^.

CTLA-4 and PD-1/L1 inhibitors have become a standard treatment of advanced malignancy including melanoma, lung cancer, and bladder cancer among others (Table 1). A significant minority of patients with metastatic disease will achieve a “durable remission” from these agents and remain free of cancer progression for years. Because of this, ICPIs are being used as palliative therapy for incurable metastatic disease and are often replacing less-effective conventional chemotherapy. An emerging area of research is the use of ICPIs in the adjuvant setting to improve the cure rate of earlier-stage disease.

**Table 1:** Food and Drug Administration-approved immune checkpoint inhibitors

Drug	Trade name	Target	Indications
Ipilimumab	Yervoy (2011)	Cytotoxic T-lymphocyte antigen 4	Melanoma
Nivolumab	Opdivo (2014)	Programmed cell death-1	Melanoma Non-small-cell lung carcinoma Renal cell carcinoma Hepatocellular carcinoma Classic Hodgkin’s lymphoma Squamous cell carcinoma of head and neck Urothelial carcinoma Colorectal cancer with microsatellite instability or mismatch-repair deficiency
Pembrolizumab	Keytruda (2014)	Programmed cell death-1	Melanoma Non-small-cell lung carcinoma Classic Hodgkin’s lymphoma Squamous cell carcinoma of head and neck Urothelial carcinoma Gastric cancer Solid tumors with high microsatellite instability or mismatch-repair deficiency
Atezolizumab	Tecentriq (2016)	Programmed cell death ligand-1	Non-small-cell lung carcinoma Urothelial carcinoma
Avelumab	Bavencio (2017)	Programmed cell death ligand-1	Merkel cell carcinoma Urothelial carcinoma
Durvalumab	Imfinzi (2017)	Programmed cell death ligand-1	Urothelial carcinoma

ICPIs have also been associated with serious immune-related adverse events due to over-activation of the immune system and can affect any organ but most commonly the gastrointestinal tract, liver, endocrine glands, and skin^4^. The pathophysiology of these immune-related adverse events has not been fully elucidated, and it is unclear why certain organ systems are affected more than others. The incidence and severity of ICPI-related side effects are generally similar across tumor types. As the use of ICPIs in oncology continues to increase, ICPI-associated colitis and hepatitis will be more frequently encountered by gastroenterologists and hepatologists. This review will focus on the diagnosis and management of immune checkpoint inhibitor-associated colitis and hepatitis, as well as compare ICPI-related toxicities to sporadic inflammatory bowel disease and autoimmune liver disease.

## Grading of immune-related toxicities

The severity of immune-related toxicities is most often graded using NCI Common Terminology Criteria for Adverse Events (CTCAE), Version 5.0 (Table 2)^5^. The accurate description of the toxicity grade is important as it dictates the treatment of the immunotoxicity and also guides when to potentially restart the ICPI therapy. PD-1 inhibitors are less frequently associated with high-grade toxicities as compared with CTLA-4 inhibitors. For example, in patients with metastatic melanoma, the incidence of adverse events with nivolumab (PD-1 inhibitor) was 82.1%, with 16.3% being grade 3–4 toxicities, compared with 86.2% and 27.3%, respectively, with ipilimumab (CTLA-4 inhibitor)^6,7^. Furthermore, the ipilimumab group had 14.8% treatment-related adverse events of any grade that led to discontinuation of the study drug compared with 7.7% in the nivolumab group^6^.

**Table 2:** Common Terminology Criteria for Adverse Events (CTCAE), Version 5

Grade	1	2	3	4	5
**Diarrhea/colitis**	Increase of < 4 stools per day over baseline (or mild increase in ostomy output compared with baseline) without colitis symptoms	Increase of 4–6 stools per day over baseline (or moderate increase in ostomy output compared with baseline) and/or colitis symptoms limiting instrumental ADLs	Increase of > = 7 stools per day over baseline (or severe increase in ostomy output compared to baseline), colitis symptoms interfering with ADLs; incontinence; hospitalization indicated; limiting self-care ADL	Life-threatening consequences (e.g., perforation, hemodynamic instability); urgent intervention indicated	Death
**Hepatitis**	AST/ALT < 3x ULN and/or total bilirubin <1.5x ULN	AST/ALT 3–5x ULN and/or total bilirubin >1.5 to ≤ 3x ULN	AST/ALT > 5–20x ULN and/or total bilirubin 3–10x ULN	Decompensated liver function, AST/ALT > 20x ULN, and/or total bilirubin >10x ULN	Death

*AST* Aspartate Transaminase, *ALT* Alanine Transaminase, *ULN* upper limit of normal

## ICPI colitis

Diarrhea is the most common symptom of ICPI-induced colitis; other symptoms may include abdominal pain, hematochezia, weight loss, fevers, nausea, and vomiting. Rare but serious complications of intestinal perforation and even death have been associated with ICPI-induced colitis or enterocolitis. For example, the incidence of colonic perforation in studies of ipilimumab ranged from 1–1.5% among patients with melanoma^2,8^ to 6.6% among patients with renal cell carcinoma^7^. A 1.1% mortality rate from complications of ipilimumab-induced enterocolitis has been reported^9^. Prompt identification of immune-related colitis can be challenging as there are other potential causes of diarrhea and the timing of onset and severity of immune-related colitis are so variable. However, early diagnosis is important both to prevent complications from persistent or worsening colitis and also to minimize the duration of ICPI therapy interruption, provided that the patient is a candidate to restart an ICPI (see “Resumption of ICPI therapy” below).

Gastrointestinal immune-related adverse events are commonly associated with anti-CTLA-4 therapy, and colitis tends to be the first immune-related adverse event leading to discontinuation of anti-CTLA-4^7,10^. Across 14 phase I–III trials of ipilimumab used for treatment of metastatic melanoma, approximately one-third of patients suffered from gastrointestinal immune-related adverse events^11^. The timing of colitis after anti-CTLA-4 therapy is variable, but generally occurs within weeks to a couple months after the initiation of therapy, though infrequently can occur even up to a year after the therapy has been discontinued. The time of colitis onset following the last dose of ipilimumab ranged from 0 to 59 days, with a median time of onset of 11 days^2,8^. The incidence and severity of gastrointestinal toxicity is dose-dependent, as patients receiving 0.3, 3, or 10 mg/kg of ipilimumab experienced incidences of grade 3 or 4 gastrointestinal immune-related adverse events of 0%, 3%, and 15%, respectively^2,12^.

Colitis is typically more frequent and severe with combination immunotherapy. The incidence of diarrhea/colitis in patients with metastatic melanoma who received a combination of nivolumab and ipilimumab was 56%, of whom 17% had grade 3 or 4 toxicity^6,7^. Moreover, the onset of grade 3 to 4 toxicities associated with combination therapy typically occurred earlier in the treatment course compared to monotherapy with either agent. There are currently no effective prophylactic regimens for ICPI colitis; in a randomizd controlled trial, budesonide did not decrease the rate of grade ≥ 2 colitis in patients with melanoma receiving ipilimumab^9^.

The work up for ICPI-associated diarrhea/colitis of grade 2 and above includes a complete blood count (CBC), comprehensive metabolic panel, thyroid-stimulating hormone (TSH), erythrocyte sedimentation rate (ESR), and C-reactive protein (CRP)^13,14^. Screening tests for hepatitis B and tuberculosis should be obtained in anticipation of the possible need for infliximab therapy. Stool samples should be sent for Clostridium difficile testing and bacterial culture or multiplex nucleic acid amplification testing (NAAT) for GI pathogens. If multiplex NAAT is not available, testing for ova and parasites and other pathogens can be considered on an individual basis^13^. There are no specific laboratory tests to diagnose immune-related toxicity; however, an elevated fecal lactoferrin or fecal calprotectin may help point to an inflammatory cause^15^.

Flexible sigmoidoscopy or colonoscopy with biopsies is recommended for patients with persistent grade 2 diarrhea and patients with grade 3–4 diarrhea^2,7^. If there are any new upper GI symptoms including oral ulceration, dysphagia, odynophagia, or epigastric pain, upper endoscopy is also recommended, as toxicities can be infrequently confined to the upper GI tract^8,16^.The typical endoscopic findings of ICPI-associated colitis range from normal to those seen in inflammatory bowel disease, including loss of vascular pattern, exudates, granularity, friability, and ulcerations. These changes are typically but not always continuous^17^. The presence of ulceration on endoscopy or a higher endoscopic Mayo score has been associated with a higher likelihood of steroid-refractory colitis requiring infliximab therapy^13,17^. It is important to note that a normal endoscopic evaluation does not rule out the presence of inflammation, and therefore biopsies should be obtained regardless of the macroscopic appearance of the colonic mucosa^8,17,18^. In contrast to the similarities in endoscopic findings between IBD and ICPI colitis, the histopathologic findings are typically different. The most common reported histopathologic findings include neutrophilic inflammation (intraepithelial neutrophils, cryptitis, and crypt microabscesses), increased crypt epithelial cell apoptosis with crypt atrophy and dropout, and a predominantly lymphocytic inflammatory infiltrate in the lamina propria^2,18–20^. Another observed pattern of inflammation resembles lymphocytic colitis with increased intraepithelial lymphocytes and a mononuclear infiltrate in the lamina propria with minimal to no evidence of neutrophilic inflammation^19^. Notably, features of chronic inflammation (crypt distortion, basal lymphoplasmacytosis, and left-sided Paneth cell metaplasia) are absent, though crypt irregularities were described in 40% of patients with ipilimumab-associated colitis^20^. Abdominal computed tomography should also be considered in patients with grade 2 or greater toxicities, particularly in patients with fever, bloody stool, or abdominal pain, to assess for toxic megacolon, exclude perforation, and evaluate for other etiologies for the symptoms^2,7^. Repeat endoscopy is favored in those who do not respond to immunosuppression or to document complete remission of ICPI-associated colitis, particularly in cases where this would allow for resumption of ICPI therapy^13^. Fecal calprotectin may be an option to monitor disease activity in addition to or in lieu of repeat endoscopy^21^, although this remains a topic in need of further investigation.

## Treatment of ICPI colitis

**Fig. 1:**
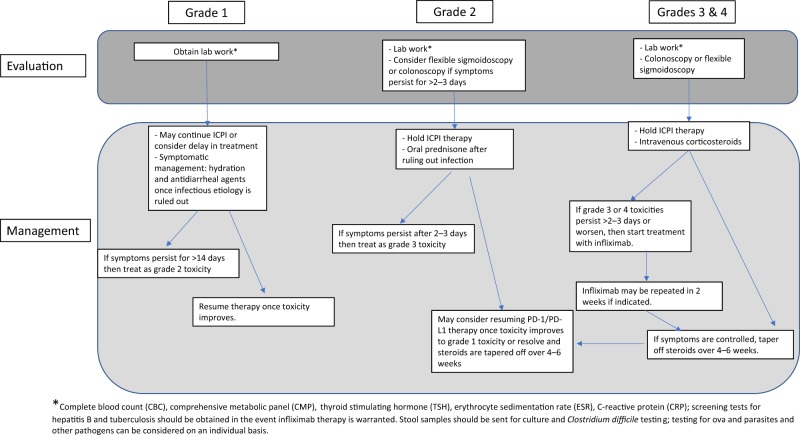
This flow diagram depicts a basic approach for evaluation and management of a patient with suspected ICPI colitis based on the severity of their symptoms as defined by Common Terminology Criteria for Adverse Events v5.0

Grade 1 ICPI colitis may be managed by the medical oncologist without extensive evaluation or gastroenterology consultation^13,14^ (Fig. 1). Anti-diarrheal agents such as loperamide may be used for symptomatic relief, and most patients can safely continue ICPI therapy. However, they should be closely monitored for dehydration and symptoms of worsening colitis. For grade 2 colitis, ICPI treatment should be held and oral or IV corticosteroids should be administered with a starting dose equivalent to 1 mg/kg/day of methylprednisolone. After symptoms are adequately controlled, the steroid should be slowly tapered over at least 4–6 weeks, given the high risk of recrudescence. If marked symptom improvement is not observed after 2–3 days of steroid therapy, the steroid dose may be increased to an equivalent dose of 2 mg/kg/day of methylprednisolone. Alternatively, a single dose of infliximab 5 mg/kg may be considered with a second dose 2 weeks later if there is evidence of ongoing colitis. It is important to note that CTLA-4 inhibitors should be discontinued permanently for grade ≥ 2 colitis, while PD-1/PD-L1 inhibitors may be restarted after grade 2–3 colitis once symptoms resolve or improve to grade 1.

Patients with grade 3–4 toxicities typically require hospitalization and should start treatment with 2 mg/kg/day of IV methylprednisolone, which should be continued until symptoms have dramatically improved. At this point, the IV corticosteroid can be converted to an oral corticosteroid with a slow taper over at least 4 weeks. Infliximab should be strongly considered if symptoms are not controlled after 2 days of high-dose IV steroids (Fig. 1). If patients are refractory to infliximab (e.g., inadequate response after two doses 2 weeks apart) or if it is contraindicated, then vedolizumab could be considered^13^. In a recent case series of seven patients treated with vedolizumab for corticosteroid-dependent or refractory ICPI-induced enterocolitis, all but one patient achieved complete remission with normalization of fecal calprotectin with treatment^21^.

## Hepatic complications

Although less frequent than ICPI diarrhea/colitis, ICPI-associated hepatotoxicity is also not uncommon. The most common form of ICPI-associated hepatotoxicity is hepatitis, which is marked by hepatocellular injury as defined by elevations in serum aminotransferases (ALT and AST) with or without elevations in serum bilirubin (Table 2). In clinical trials of these agents, hepatitis occurred in fewer than 5–10% of patients treated with a single ICPI^6,22^ of whom 1–2% experienced grade 3–4 hepatitis. However, grade 3–4 ALT elevations were observed in ∼20% of patients receiving the combination of ipilimumab at 3 mg/kg and nivolumab at 1 mg/kg^6^, consistent with the finding that combination CTLA-4 and PD-1/PD-L1 blockade is associated with more frequent and more severe immune-related toxicities in general.

ICPI-associated hepatitis is most often asymptomatic, thus a liver panel should be measured at baseline and before each treatment cycle. The typical timing of ICPI-associated hepatitis is in the range of 6–14 weeks following treatment initiation^7^. In some cases, ICPI-associated immune toxicities can be quite delayed, appearing many months following treatment initiation or even after treatment completion.

## Treatment of ICPI hepatitis

**Fig. 2:**
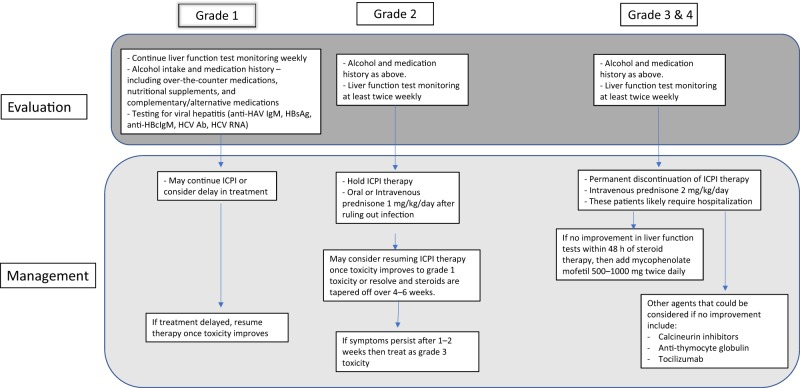
This flow diagram depicts a basic approach for evaluation and management of a patient with suspected ICPI hepatitis based on the severity of their symptoms as defined by Common Terminology Criteria for Adverse Events v5.0

For mild grade 1 elevations in AST or ALT (Table 2), the scheduled ICPI dose can be administered with continued liver test monitoring (Fig. 2). A careful alcohol intake and medication history (including over-the-counter medications, nutritional supplements, and complementary/alternative medications) should be performed to evaluate for drug-induced liver injury. Additionally, testing for viral hepatitis (anti-HAV IgM, HBsAg, anti-HBcIgM, HCV Ab, and HCV RNA) should be performed. Although testing for serologic markers of autoimmune liver disease has been suggested^7^, they are typically negative among patients with ICPI-associated hepatitis^23,24^ and thus should not be used to establish or exclude the diagnosis of ICPI-associated hepatitis. For bilirubin elevation with or without transaminase elevation, ultrasonography should be performed to evaluate for biliary obstruction; if the ultrasound is normal, magnetic resonance cholangiopancreatography (MRCP) should be considered.

For grade 2 elevations in liver tests (Table 2), ICPI therapy should be held and liver tests should be monitored at least twice per week. If the transaminase elevations persist beyond 1–2 weeks or rise to grade 3–4 in severity, then corticosteroid therapy should be initiated; this should not be delayed for the results of serologic testing if there is no other obvious cause from history. If the liver tests improve, then ICPI therapy can be resumed once the steroid dose has been slowly tapered to ≤10 mg/daily of prednisone (or equivalent) and liver tests have returned to baseline. For grade 3–4 hepatotoxicity, ICPI therapy should be permanently discontinued and steroids should be promptly initiated at an equivalent dose of 1 mg/kg/day of oral (grade 3) or 2 mg/kg/day of IV (grade 4) of methylprednisolone.

Most cases of ICPI-associated hepatitis respond to corticosteroid therapy, although a prolonged corticosteroid taper over 6–8 weeks or more may be required. If the liver test abnormalities do not begin to improve within a week of initiating corticosteroid therapy, then the recommendation is to add mycophenolate mofetil (MMF) at a dose of 500–1000 mg twice daily, though the published evidence for this recommendation is limited^25^.

Most cases of ICPI-associated hepatitis resolve completely with corticosteroid therapy, occasionally requiring the addition of MMF. Those who do not respond to combined corticosteroid and MMF therapy should be promptly referred to a medical center with hepatology consultation. While there is currently no established role of liver biopsy in the evaluation of patients with severe ICPI-associated liver injury, it may be considered in cases where there is diagnostic uncertainty regarding the cause of the liver injury. One case series of 11 patients who underwent liver biopsy for ipilimumab-associated hepatitis showed histopathologic evidence of active hepatitis in nine^24^. Among these nine patients, six displayed a pattern of panlobular hepatitis and three a pattern of centrilobular (zone 3) hepatitis. Of the remaining two patients, one had a pattern of cholangitis with a predominantly portal inflammatory infiltrate and the other had features of steatohepatitis indistinguishable from those of nonalcoholic steatohepatitis. A second case series included three liver biopsies, two of which also showed a pattern of panlobular hepatitis and the third a pattern of cholangitis and portal inflammation^23^.

There is currently little evidence to guide the management of ICPI-associated hepatitis refractory to combined corticosteroid/MMF therapy. Tumor necrosis factor-alpha inhibitors such as infliximab have demonstrated efficacy for several other immune-related ICPI toxicities, but are not currently recommended for the treatment of ICPI-associated hepatitis as they have been associated rarely with several forms of idiosyncratic drug-induced hepatotoxicity^26^. Because of this, the efficacy and risk of hepatotoxicity with TNF alpha inhibitors for the treatment of ICPI-associated hepatitis have not been defined. Other agents that could be considered include calcineurin inhibitors such as tacrolimus or cyclosporine A, anti-thymocyte globulin^27,28^, and tocilizumab^29^.

While ICPI-associated hepatotoxicity is in the great majority of cases characterized by hepatocellular injury, there have also been reports of cholestatic liver injury associated with ICPIs. Cholestatic liver injury is characterized by elevations in serum alkaline phosphatase, with or without serum bilirubin elevation. The histopathologic findings in three reported cases included varying degrees of bile duct injury and ductopenia, including one in a pattern consistent with vanishing bile duct syndrome^30^, though no biliary imaging was reported. A second series of 91 patients treated with nivolumab for metastatic non-small cell lung cancer reported that three developed a cholestatic pattern of liver injury with negative markers of autoimmune liver injury and normal serum IgG4 levels^31^. CT scans demonstrated marked extrahepatic biliary dilation with normal intrahepatic bile ducts and no evidence of biliary obstruction by endoscopic ultrasound or cholangiopancreatography (magnetic resonance or endoscopic). Five of the six patients in these two series received steroid therapy; although a small number, it is worth noting that all of them showed fair to poor responses to treatment with steroids or with steroids combined with MMF.

## Resumption of ICPI therapy

The decision to resume ICPI therapy is challenging as many factors need to be considered. On one hand, patient outcomes are seemingly not worsened by the use of immunosuppression to treat ICPI colitis^10,32^. Retrospective studies suggest that time to treatment failure is similar for patients treated with immunosuppressive agents when compared with patients who did not receive immunosuppression for immune-related adverse events. This would suggest that patients with a good initial response may not need to be rechallenged, as these responses are typically durable. However, clinical factors like a slow response to ICPI therapy, short duration of ICPI therapy, or rapid resolution of colitis/hepatitis may push the treating clinician to rechallenge with an ICPI. This requires a thorough discussion among the patient, oncologist, and gastroenterologist.

Patients who suffer from grade 2 or higher diarrhea/colitis from CTLA-4 agents should not be rechallenged with anti-CTLA-4^2,4,13^. However, it may be reasonable to switch to anti-PD-1/PD-L1 agents after the GI symptoms resolve or improve to grade 1. For example, retrospective data indicate that metastatic melanoma patients who suffered from a serious anti-CTLA-4-related adverse event (defined as grade 3–4 and/or requiring systemic immunosuppression) were able to be safely started on an anti-PD-1 agent^4,33^. Similarly, some patients with grade 2–3 diarrhea/colitis after anti-PD-1/PD-L1 therapy can be successfully rechallenged with an ICPI after symptoms resolve or improve to grade 1. In another study of 38 non-small cell lung cancer patients who developed ICPI-related adverse events requiring corticosteroid therapy, 50% of patients were restarted on their initial anti-PD-1 or PD-L1 therapy with no subsequent ICPI-related adverse events. However, 26% of patients had a new immunotoxicity and 24% had recurrence of their initial immune-related adverse event with re-initiation of therapy^34^. Therefore, the decision to rechallenge patients with ICPI-associated colitis with anti-PD-1/PD-L1 is highly individualized, and there are few prospective data from clinical trials to guide this decision. Several factors that should be considered include how well the malignancy was controlled at the time of toxicity, how quickly the toxicity was resolved with the treatment, the amount of previous ICPI therapy, and the patient’s performance status. For example, a patient with high tumor burden who is symptomatic from cancer and who only received a few ICPI treatments may have more potential benefit from ICPI rechallenge than an asymptomatic patient with minimal disease who developed ICPI toxicity after several months of ICPI therapy.

Similar considerations apply to rechallenging patients with ICPI-associated hepatitis. As discussed above, ICPI therapy can be resumed for mild (grade 2) hepatitis once liver tests have returned to baseline and steroids have been tapered to an equivalent dose of ≤10 mg of prednisone daily. Patients with grade 3–4 hepatitis from ICPI therapy should not be treated with either anti-CTLA-4 or anti-PD-1/PD-L1.

## ICPI-associated colitis, sporadic inflammatory bowel disease, and the gut microbiome

The phenomenon of ICPI-associated immune toxicities raises the question of whether these toxicities can help inform our understanding of the pathogenesis of “sporadic” autoimmune intestinal and liver diseases, and conversely whether we can apply our understanding of the sporadic autoimmune diseases to the treatment of ICPI-associated immune toxicities. While the endoscopic appearance of ICPI-associated colitis is very similar to that of inflammatory bowel disease (IBD), the histopathologic findings typically differ as discussed above. It is not yet known whether genetic polymorphisms associated with increased risk of IBD are also associated with increased risk and/or severity of ICPI-associated colitis. If so, this could potentially be used to help estimate the risk of ICPI colitis prior to treatment initiation. Some of these genetic polymorphisms associated with IBD risk are thought to affect the immune sensing of enteric bacteria. Intriguingly, there have been reports describing an association between the composition of the gut microbiome and risk for developing ICPI-associated colitis^35,36^. One of these also reported that the intestinal microbiome composition associated with an increased risk of colitis was also associated with cancer treatment response^35^. A recent publication in a mouse model of dextran sulfate sodium chemical colitis augmented with anti-CTLA-4 and vancomycin treatment found that the administration of *Bifidobactrium* reduced the severity of colitis^37^. Future prospective studies will have to be performed to assess whether the modulation of the gut microbiome in humans affects the risk of ICPI-associated immune toxicities.

A related question is whether ICPI therapy can be safely used in patients with pre-existing inflammatory bowel disease. Phase III trials of ICPI therapy have excluded patients with significant pre-existing autoimmune disorders. Two retrospective studies with ipilimumab^33^ or anti-PD-1^38^ found that 0/5 and 2/6, respectively, patients with IBD flared on ICPI therapy. Both of the flares responded to immunosuppression. It should be noted that among the 12 patients, four had a history of UC with colectomy and the remainder apparently had well-controlled IBD. Whether patients with more active inflammatory bowel disease tolerate ICPI therapy as well remains an open question and at this time will have to be decided on an individual basis.

## ICPI-associated hepatotoxicity and autoimmune liver disease

T cells are thought to play a major role in the pathogenesis of autoimmune liver diseases such as autoimmune hepatitis, and so it is tempting to speculate that the mechanism of ICPI-associated hepatitis might be similar to that of autoimmune hepatitis. However, as noted above, serologic markers associated with autoimmune hepatitis are typically negative in cases of ICPI-associated hepatotoxicity. There is no published evidence on the safety of ICPI therapy in patients with pre-existing autoimmune liver disease, though studies on the safety of ICPI therapy in patients with other autoimmune disorders suggest that patients with well-controlled autoimmune liver disease may be candidates for ICPI therapy, particularly if there are no other therapeutic options for their malignancy. Molecular characterization of the liver-infiltrating inflammatory cells (e.g., expression of cell surface markers and single-cell RNA-seq) in these two disorders would be a useful first step in determining whether they share common pathogenic mechanisms.

## Conclusions

The field of cancer immunotherapy is rapidly developing with many new immunomodulating agents in the pipeline. The expanding use of immune checkpoint inhibitors for many common cancers will result in an increase in the number of patients with immune-related side effects. Patients with severe or steroid-refractory immune-related gastrointestinal/hepatic side effects will require collaboration between the oncologist and gastroenterologist/hepatologist.

Treatment of severe ICPI-related colitis and hepatitis should include prompt discontinuation of immunotherapy and initiation of high-dose corticosteroids, with prompt escalation to other agents such as infliximab (colitis) or MMF (hepatitis) for patients who do not rapidly respond to steroids. The resumption of immunotherapy is an individualized decision that involves several factors including patient preference, the severity and onset of immune toxicity, performance status of the patient, status of the tumor, and how quickly the side effect is resolved with the treatment. More research is needed to identify biomarkers to predict the risk of immune-mediated toxicity, reduce the risk of immune-mediated toxicity, and to develop more efficacious treatment strategies for toxicity refractory to high-dose steroids.

### Study highlights

#### What is current knowledge

- Immune checkpoint inhibitor therapy is associated with GI adverse events.
- Typical clinical presentations of ICPI-associated colitis and hepatitis.
- Consensus recommendations regarding treatment of ICPI-associated GI toxicities.

#### What is new here

- Comparison of ICPI-associated colitis and hepatitis with idiopathic IBD and autoimmune liver disease.
- Brief review of gut microbiome and ICPI-associated colitis.
- Considerations for rechallenging patients with history of ICPI toxicity, as well as treating patients with idiopathic IBD or autoimmune liver disease with ICPIs.
