# Correction: Immune Checkpoint Inhibitor-Associated Colitis and Hepatitis

**DOI:** 10.1038/s41424-018-0072-x

**Published:** 2018-11-14

**Authors:** Haritha G. Reddy, Bryan J. Schneider, Andrew W. Tai

**Affiliations:** 1 Rogel Cancer Center, Division of Hematology/Oncology, Department of Internal Medicine, University of Michigan, Ann Arbor, MI USA; 2 Division of Gastroenterology, Department of Internal Medicine, University of Michigan, Ann Arbor, MI USA; 3 Department of Microbiology & Immunology, University of Michigan, Ann Arbor, MI USA; 4 Medicine Service, VA Ann Arbor Healthcare System, Ann Arbor, MI USA

Correction to: *Clinical and Translational Gastroenterology* 10.1038/s41424-018-0049-9; published online 19 September 2018

The original version of this Article contained an error in the spelling of colitis in the article title. It was incorrectly spelt as “colits”. This has now been corrected in both the PDF and HTML versions of the Article.

